# Modifying Psychiatric Approaches to Respond Better to Indigenous People in Maine (North America)

**DOI:** 10.1192/j.eurpsy.2022.371

**Published:** 2022-09-01

**Authors:** L. Mehl-Madrona, B. Mainguy, L. Sockabasin

**Affiliations:** 1 Wabanaki Public Health and Wellness, Public Health, Bangor, United States of America; 2 Coyote Institute, Education, Ottawa, Canada

**Keywords:** Indigenous people, Narrative psychotherapy, Ceremony and Ritual, Power Threat Meaning Framework

## Abstract

**Introduction:**

Psychiatry has typically underserved indigenous people and immigrants. Indigenous people have different ways of viewing mind and mental health and conventional Euro-American psychiatry has not always acknowledged that.

**Objectives:**

We wanted to modify conventional psychiatric approaches to better serve our indigenous population. We worked together to determine what that would be, gained feedback from indigenous patients and practitioners, and wanted to describe what we learned in an autoethnographic fashion.

**Methods:**

We engaged each other, indigenous practitioners within the community and indigenous patients in an ongoing discussion of how psychiatry should change to be relevant to indigenous people. We monitored our own process in an autoethnographic fashion.

**Results:**

1. The typical DSM (Diagnostic and Statistical Manual) or ICDA (International Classification of Diseases) categories were difficult to apply to the lives of many of these patients, given the high levels of trauma both experienced and transmitted epigenetically (inter-generational trauma). A power-threat-meaning framework appeared to be a more useful adjunct to these classifications along with trauma-informed perspectives. 2. Conventional cognitive behavior therapy was less accepted given its emphasis on rational thinking, while narrative approaches were more successful, given the widespread uses of stories and storytelling in these cultures and the emphasis on relationship as more important than rationality. 3. Trained peer counselors were very helpful. 4. Bringing culture (language, songs, ceremonies, elders, arts) into treatment was highly desirable.

**Conclusions:**

Psychiatric services to indigenous and immigrant communities should focus on empowerment through community-based, participatory methods, facilitating local problem solutions, and involving traditional elders, local government, and other stakeholders.

**Disclosure:**

No significant relationships.

